# The Water Footprint of Hydropower Production—State of the Art and Methodological Challenges

**DOI:** 10.1002/gch2.201600018

**Published:** 2017-06-06

**Authors:** Tor Haakon Bakken, Ånund Killingtveit, Knut Alfredsen

**Affiliations:** ^1^ Department of Hydraulic and Environmental Engineering Norwegian University of Science and Technology 7491 Trondheim Norway; ^2^ SINTEF Energy Research 7465 Trondheim Norway

**Keywords:** hydropower, multipurpose, reservoirs, water consumption, water footprint

## Abstract

This paper reviews published estimates of water consumption from hydropower production and the methodologies applied. Published values range from negative to more than 115 000 m^3^ MWh^−1^. Most gross water consumption rates are in the range 5.4–234 m^3^ MWh^−1^, while most net values are in the range 0.2–140 m^3^ MWh^−1^. Net values are often less than 40% of the gross values, sometimes only 1% of the gross water consumption estimates. The extremely wide range in estimates is explained by an inconsistent methodology and the very site‐specific nature of hydropower projects. Scientific challenges, such as allocation from multipurpose reservoirs, and spatial assignments in river basins with several hydropower plants, affect the results dramatically and remain unresolved. As such, it is difficult to propose “typical values” for water consumption from hydropower production. This paper points out directions of research in order to prepare a consistent and improved methodology for the calculation of water consumption from hydropower projects. This should take into account the role of reservoirs in the provision of a large range of water services, as well as providing regulated power to the energy system.

## 1 Introduction

A rapidly growing population, economic development, and increasing consumption lead to a massive use of the Earth's resources.^1^ Many countries and regions experience water stress and ecological degradation of aquatic ecosystems, which is expected to further increase and accelerate with climate change.^2^, ^3^, ^4^ Approximately two‐thirds of the anthropogenic emissions of greenhouse gases causing climate change originate from the energy sector, i.e., in the combustion of coal, oil, and gas in the conversion of fossil fuels into electricity and heat.^5^ As such, the energy sector is a key player in the transition into a low‐carbon society. In order to reach such a target, there is a need to increase the share of low or no carbon energy production, which has boosted large‐scale investments in solar, wind, and hydropower.^5^

There is a growing understanding of the close relation between water and energy, sometimes entitled the water–energy nexus.^6^, ^7^, ^8^, ^9^, ^10^ Water is highly needed in the development of energy resources, and access to energy is a prerequisite in the provision of water services. According to ref. 10, primary energy production and power generation accounted for roughly 10% of total worldwide water withdrawals and around 3% of total water consumption in 2014, which corresponds to withdrawals of around 400 billion m^3^ annually. Several regions now experience water stress,^11^ and access to water might constrain the further development of energy sources.

Production of hydropower is exclusively dependent on the availability of the local water resources, and reduction in the available water will immediately reduce the power production. Among the world's 45 000 registered large dams and reservoirs,^12^ ≈75% are classified as single purpose, meaning that they are used exclusively for one specific purpose.^[13]^ The most common single purpose is irrigation (47%), followed by hydropower (19%), water supply (11%), flood control (9%), and the remaining recreation, navigation, and “unclassified.” Among the multipurpose reservoirs, serving two or more functions, which is ≈25% of the registered dams, irrigation is also the most frequent purpose. 61% of the registered multipurpose reservoirs have irrigation as one of several functions, followed by flood control (50%), water supply (44%), hydropower production (40%), recreation (30%), and navigation (6%).

The world's reservoirs are expected to play an even more important role for the society in the future. Climate change might reduce the available water in arid areas of the world, increase the runoff in wet areas, and change the seasonality. Large developments of intermittent power sources, such as wind and solar power, can also increase the need for flexible energy production,^10^ served by for instance by hydropower with reservoirs.^14^

This paper aims at:

1. Reviewing published estimates of water consumption from hydropower production.
2. Presenting the methodology applied, identify shortcomings and propose improvements.
3. Discussing the complexity of reservoirs and river regulations, and how methodological choices will affect the water consumption rates.

For the hydropower sector there are at least two important aspects related to the topic water consumption from power production. First, high water consumption rates represent a potential reputational risk to the sector, as the whole hydropower sector can be stamped as being “large water consumers.” Second, the establishment of upstream reservoirs might reduce the inflow to the downstream power producers. Upstream reservoirs will usually increase evaporative losses, and the higher degree of regulation might enable larger water withdrawals to for instance irrigation, both effects potentially posing a financial risk to downstream operations.

## 2 State of the Art

### 2.1 Clarification of Terms

A number of different terms and definitions are used to describe the use and consumption of water, and a consistent terminology is not in place. US Geological Survey defines water withdrawal as water removed from the ground or diverted from surface‐water sources for use.^15^ This includes both a consumptive and a nonconsumptive part. Water consumption is the part of the water that is evaporated, transpired, incorporated into products or crops, consumed by humans or livestock, and not returned back to the river basin. The nonconsumptive part of the water withdrawal is the water that is returned back to the river basin. Pfister et al.^16^ define water consumption as the part of the freshwater which is not released back to the original watershed; primarily due to evaporation and product integration.

Olsson^8^ adopts a slightly different interpretation of the term water consumption by arguing that water is consumed when the control over the water is lost, e.g., due to evaporation. The Water Footprint Network^17^ uses the term “water footprint” and defines the water footprint of a product (a commodity, good, or service) as the total volume of freshwater used to produce the product, summed over the various steps of the production chain. This definition refers to a life‐cycle perspective of the production of a commodity, good, or service. In the context of energy production, it does not seem to be a clear differentiation between the terms “water footprint” and “water consumption.” Water scarcity footprints, as defined by Scherer and Pfister,^18^ takes into account also the potential positive effect of the water availability due to the regulation. The outcome of water scarcity footprint assessments in the context of hydropower is that reservoirs potentially alleviate water scarcity despite their high water consumption.

Water consumption allocation is a term used in relation to water consumption from multipurpose reservoirs. Water consumption allocation is an exercise of distributing the consumed water between all functions and stakeholders benefitting from the regulation. This is relevant for instance in those cases reservoirs serve several functions, as multipurpose reservoirs do. Water consumption allocation is a field of science that has undergone very limited research, and should not be mixed up with water allocation, which is about allocating the (remaining) water available for use between potentially competing interests, e.g., drinking water supply, irrigation, navigation, and power production.

### 2.2 Published Values on Water Consumption from Hydropower Production

The published values on water consumption from hydropower production vary in their spatial extent, from global averages, regional assessments, and to single plant values. The studies are also published in various formats, ranging from peer‐reviewed journals, technical reports, and master theses. This might also affect the quality and precision of the water consumption estimates. **Table** **1** presents studies that are considered “key publications” within this field of science, based on to what extent they have given new insight into the topic, how much attention they have gained in the hydropower sector, and their scientific quality. We underline that these studies do not represent an exhaustive list of publications in this field of science.

**Table 1:** Key publications on water consumption estimates from hydropower production and range of published values. Values given in nonitalicized text are water consumption estimates based on net evaporation values, while values in italic and parentheses are gross water consumption values. The column “Geographical coverage (# of power plants)” describes which region the water consumption values are calculated from, and the approximate number of power plants included in the study (in parentheses). All values are given in m^3^ MWh^−1^

Publication	Min. value	Max. value	Ave. value	Geographical coverage (# of power plants)	Comment on methodology
Ref. 19	(*0.04*)	(*209*)	(*5.4*)a)	California, USA (≈100)	Gross values, no allocation
Ref. 20	N.A.	N.A.	(*68*)	USA (≈120)	Gross values
Ref. 21	N.A.	N.A.	(*80*)	Global	Gross values
Ref. 3	(*0.04*)	(*209*)	N.A.	California, USA (≈100)	Review study
Ref. 22	1.7 (*2.9*)	70.9 (*117.0*)	9.8 (*21.8*)	New Zealand (17)	Both gross and net values reported, no allocation
Ref. 23	(*1.08*)	(*3045.6*)	(*244.8*)	Global (35)	Gross values, no allocation
Ref. 24	0.15 (*4.5*)	0.19 (*71.1*)	0.17 (*37.8*)	Norway (2)	Both gross and net values reported, spatial allocation
Ref. 25	(*2.9*)	(*33.1*)	(*5.4*)	Three Gorges, China (1)	Gross values, multipurpose allocation between functions made
Ref. 26	(*0.004*)	(*15 424*)	(*13.0*)	China (209)	Gross values, multipurpose allocation made
Ref. 27	7 (*39*)	16 (*53*)	14	Canada (1)	Both gross and net values reported, no allocation
Ref. 28, b)	0	N.A.	37.3	USA (≈650)	Net values, multipurpose allocation made
Ref. 29	−75.6 (*3.6*)	104.4 (*417.6*)	6.1 (*39.6*)	USA (≈4000)	Both gross and net values, multipurpose allocation based on primary function
Ref. 18	0.1 (*0.3*)	115 884 (*171 220*)	140c) (*234*)	Global (≈1500)	Both gross and net values reported

^a)^ The median value is given in ref. 19

^b)^ Lake evaporation, background evaporation, and power production are given for a large number of individual reservoir‐based plants, but water consumption values are not calculated for individual plants. The given values are provided in the Supporting Information

^c)^ Pfister et al.^18^ found gross and net median values of 120.6 and 61.6 m^3^ MWh^–1^, respectively.

Water consumption values given in Table 1 are not calculated based on a consistent methodology, which makes internal comparison between hydropower projects difficult, as well as with other energy technologies. For this reason, hydropower was excluded from the comparative presentation of electricity‐generation technologies in ref. [10, p. 356], as “the amount consumed is highly site‐specific and the measurement methodology is not agreed upon.” In the following, differences in methodology and assumptions made are presented, referring to the values given in Table 1.

Water consumption from hydropower production is basically calculated as the annual evaporation volumes divided on annual power production, given for instance in m^3^ MWh^−1^. The majority of the studies published has used the gross evaporation from the reservoir surfaces as the basis. In more recent studies, water consumption based on net evaporation rates is more commonly reported (e.g., refs. 18, 22, 24, 27, 28, 29). When net evaporation rates are calculated, the evapotranspiration prior to the establishment of the reservoir is subtracted from the evaporation from the reservoir surface. Usually the long‐term annual water consumption is calculated. Ref. 25 presents year‐to‐year water consumption values, and the results show interestingly large differences between the lowest and the highest values.

Most studies including multipurpose reservoirs suffer from lack of proper allocation of the water losses. In a very few cases, allocation is carried out, but no consistent methodology has been used.^18^, ^26^, ^28^, ^30^ In all other known studies, the burden of the water losses is allocated to power production alone, under the assumption that this is the only or the main function of the reservoir.

A related challenge to allocation is the spatial assignments that should be made in cascaded river basins with several hydropower plants which all benefits from one or more reservoirs. This is a methodological problem that is hardly discussed in the scientific literature, and water consumption values are simply calculated by assigning all the water losses to the hydropower plant closest to the reservoir. Except in very few cases,^24^, ^28^ run‐of‐the‐river plants are assigned no losses.

All studies assume that the water consumption from the operational phase by far dominates the water consumption from the construction and decommission, and hence neglect the water consumption from these phases. Ref. 24 is the only known study that has investigated this assumption, and concluded that it is probably a reasonable assumption to neglect water consumption from construction and decommission, maybe except in cases located in cold climate regions. The conclusion is drawn based on two case studies in Norway, with generally very low total net water consumption values (Table 1), where the construction phase contributed with 32% and 9% of the total water losses.

All the values presented in Table 1 are given without characterization, i.e., without any kind of impact assessment based on the state of the local or regional water resources.

## 3 Dilemmas Related to the Applied Methodology

### 3.1 Gross versus Net Evaporation Rates

It seems to be a growing support to the *net* water consumption methodology as the proper approach in the calculation of the “true” water consumption from hydropower production (e.g., refs., 18, 26, 27, 28, 29,31). There are, however, clearly divergent scientific views on this, and the Water Footprint Manual^32^ prescribes using the gross evaporation values in water consumption assessments. The difference between the net and gross evaporation will vary extensively depending on climate, land use prior to inundation, and the design of the individual hydropower facility. The establishment of large reservoirs in arid regions with high evaporation rates, such as Lake Nasser in Egypt, will create very large water losses^33^ and the difference between the net and the gross losses are small. Scherer and Pfister^18^ found the net water consumption to be typically 40% lower than the gross water consumption. This is in line with the findings by Herath et al.,^22^ which reported the net water consumption to be in the range 45%–60% of the gross rates. Bakken^24^ found this ratio to be much smaller and the net rates were less than 2.5% of the gross values.

Net water consumption values can also end up being negative.^29^ Negative water consumption values would mean that there is more water available downstream after regulation compared to the situation prior to the regulation. Several reasons can explain this effect. One reason is that vegetation can have higher evapotranspiration rates than water surfaces (e.g., ref. 34). Reservoirs developed from natural lakes can also be operated with lower surface levels than during natural conditions, known as “draw‐down” reservoirs, resulting in negative net water consumption values. The operational regime will affect the evaporation losses, as the surface area will vary directly depending on water level. Data on the operation of the reservoirs and the variations in water level are generally not accounted for in these studies. Furthermore, the effect of reduced flood plains (downstream) due to regulation can lower the net water consumption values.

The most important argument for using the net evaporation rates in the calculation of the water consumption is that the use of gross evaporation values obscures the true effect of reservoirs on the local/regional water resources. Only calculations based on net evapotranspiration will inform decision makers about what changes the reservoir will introduce. One practical reason why many studies still have calculated water consumption based on gross evaporation rates can be the challenge of calculating evapotranspiration. This is particularly problematic when a large number of hydropower projects are assessed. As new global datasets on evapotranspiration and more detailed climatic data are available, parts of this challenge should be possible to overcome, even for large datasets. This might make contribute to reach a scientific consensus on whether the net or gross evaporation calculations should be applied in water consumption studies. The problem of finding reliable information about land cover prior to establishment of the reservoir is, however, a challenge that still persists.

### 3.2 Allocation between Functions in Multipurpose Reservoirs

There is no scientifically accepted method to allocate water losses from multipurpose reservoirs between all functions benefitting from the regulation. In the majority of studies carried out, hydropower is assumed to be the single or main purpose, or the problem seems to be ignored, and all the burden of the water losses is assigned the power production. The study by Pasqualetti and Kelley^30^ was, according to our knowledge, the first study that handled this methodological challenge and used economic value as the approach to distribute the water losses. Zhao and Liu^25^ used the same approach for allocation, by calculating the economic value of hydroelectricity to the total economic value of all ecosystem services provided (flood control, navigation, water supply, fisheries, and hydropower production), and from this ratio assigned a share of the water consumption to power production. Lampert et al.^28^ assigned water losses to power production from multipurpose reservoirs by adopting the same water consumption rates (m^3^ MWh^−1^) as found for single purpose hydropower reservoirs. Grubert^29^ applied a primary purpose based approach, which means that the primary purpose of the reservoir takes the full burden of the water losses. Grubert^29^ argues that other approaches, such as the volumetric approach, are not practical and it is difficult to find a single and consistent data source that can provide input data on all functions when carrying out system‐wide assessments, i.e., assessing the water consumption rates from a large number of plants within a region. Scherer and Pfister^18^ used a ranking method, which gave allocation rates from the number of functions and the specific rank number of hydropower production.

Choice of allocation method is a known problem from Life Cycle Assessment (LCA)‐based studies, and the basis for allocation in LCA‐studies is often ISO Standard 14044.^35^ According to the authors' knowledge, the study by Bakken et al.^36^ was the first of its kind that systematically tested out allocation methods as specified by ref. 35. The study tested four different allocation methods in four multipurpose reservoirs, all serving three to five functions, and concluded that volume allocation was the most feasible and robust approach. Economic allocation was found by Bakken et al.^36^ to be sensitive to selection of economic valuation method, but acknowledged that this method could be useful in those cases where the same economic valuation method was applied across all functions served by the reservoir. Kadigi et al.^37^ underline that use of economic valuation methods could underestimate the social value of water.

Zhao and Liu^25^ developed dynamic allocation factors based on the annual economic benefit for each of the functions benefitting from the multipurpose reservoir through a 10 years period. In the case of hydropower, the allocation factors varied between 38% and 76%. Similarly,^29^ presents an analysis in how the water intensity varies with choice of allocation method. The methods “Equal weighting,” “Economical valuation 1,” “Primary purpose,” and “Economical valuation 2” allocate in the range 35% to 55% of the water losses to hydropower, while “None to hydro” and “All to hydro” are in the extreme ends allocating 0% and 100% to this function, respectively. The study by Bakken et al.^36^ showed a similarly large spread in allocation ratios. The studies illustrate that choice of method will affect the assignment of losses to the hydropower production dramatically, which was also underlined by Lampert et al.^28^.

Bakken et al.^36^ underlined that the recommended volume‐based allocation method will not capture the full complexity of regulations and a hybrid of different methods might provide more reasonable and robust output. The next step in refinement of allocation method might be a methodology that takes into account elements from economic allocation, the priority of the water use from the multipurpose reservoir, as well as the degree of regulation needed by each function. The added refinements for the purpose of making the allocation “more fair” must, however, be balanced with the resources needed to collect additional data. One allocation method might be appropriate when one or a few hydropower facilities are assessed, and some on‐site data collection is acceptable. In those cases a large number of hydropower facilities are assessed, access to national or global databases might be prerequisite.

### 3.3 The Spatial Effect of a Regulation and Assignments of Losses

A difficult methodological challenge that surprisingly seems to be overlooked and hardly discussed in studies of water consumption from hydropower production is how the spatial boundaries are set in the analysis. Most studies draw the spatial boundaries around the power plant and the reservoir in its immediate vicinity. Hydropower plants are often developed in a cascade, where several downstream plants (run‐of‐the‐river plants) benefit from an upstream regulation. Lack of spatial assignments of the water losses between all hydropower plants benefitting from the regulation will lead to an unfair and too large burden on the plants in direct connection to the reservoirs, while run‐of‐the‐river plants are assigned too low values (often considered having a “zero water footprint”). This can also explain parts of the large variability on water consumption estimates published. In regional or national assessments of water consumption this problem is nonexisting as the water losses are simply summed and divided on the sum of the energy produced.

Many hydropower regulations can be very complex, exemplified by Statkraft's Ulla‐Førre development,^38^ or Agder Energi's slightly less complex development in Mandal River Basin.^31^ In these river basins some sections will have less water, and other sections more water after regulation, which also often vary throughout the year. The areas downstream the power plants typically experience an annual redistribution of the flow. Hydropower plants classified as run‐of‐the‐river plants might introduce a widening of the upstream river, turning it into a long and narrow lake. A small raise in water level due to the inlet construction can increase the water surface areas and the evaporative losses.

Reservoirs in the upper part of the river basin can also be disadvantageous and pose a risk of operation to power producers in the lower parts of the basin. Upstream regulation might enable increased irrigation water withdrawals, hence reducing the power production, by its provision of regulated flow.^39^ In such cases, it would be unfair if downstream hydropower plants should carry parts of the burden of the upstream water losses if the reservoir is not beneficial. In order to assess the role of a reservoir on the individual power plant, more detailed studies must be made before spatial allocation can be made.

A fundamental discussion related to spatial boundaries, is whether the water that evaporates from reservoirs is “truly lost.” Berger et al.^40^ studied basin internal evaporation recycling in the context of water footprinting and found river basins where more than 30% of the evaporated water returned to its basin of origin. Related is the study of Degu et al.,^41^ which concluded that large lakes/reservoirs can create their own microclimate with elevated air moisture and higher precipitation than without the presence of reservoirs. The effect of a new microclimate created by reservoirs seems in particular to be the case for areas dominated by convective precipitation.^42^ The evaporation from reservoir surfaces will introduce a spatial, and possibly temporal, redistribution of water, but should not be considered lost without further investigations.

There is no clear methodology for the spatial assignment of water losses between several hydropower plants within the same regulated system. A first step in the assessment should be to identify the influence area of the regulation, and several different approaches might be possible to apply for the distribution of losses. In ref. 24 the water losses were distributed based on energy production (MWh) and installed capacity (MW), depending on availability of data. Lampert et al.^28^ also allocated all the losses to the plant closest to the reservoir, but made a clear statement than other plants benefitted from the regulation (but received no water consumption).

Other approaches to be evaluated for spatial allocation could be allocation based on the degree of regulation. Those plants closer to the reservoir will benefit more than reservoirs located further away. Another alternative could be to take into account the order of development and the incremental effect posed by adding a new hydropower plant in a cascade. Furthermore, ownership could also be considered, as the producers controlling the release of water will have larger benefits of the regulation. Similar to the discussion on allocation from multipurpose reservoirs, there are no universally correct answers, but allocation procedures that are considered fair. Defining reasonable spatial boundaries for a water consumption assessment should be made with great care in order to capture all aspects and effects discussed in this section. The spatial boundaries are definitely wider than only one reservoir, and should possibly include the whole river basin.

### 3.4 Assessment of Impacts and Changes in the Water Availability

The impact of water consumption from reservoir evaporation is reduced flow downstream, while the positive effect of the reservoir is improved availability. Traditional impacts from hydropower regulations on the environment, such as impacts on biodiversity, local fisheries, and landscape, are not discussed in this paper. They are handled by environmental impact assessments, which are well established instruments in many countries.

The impacts due to the elevated evaporative water losses are to a limited extent assessed in published literature. There is also a scientific debate whether such an impact assessment beyond a simple volumetric calculation of water losses should be made (e.g., refs. 43, 44, 45). Scientists supporting the methods described in the Water Footprint Manual^32^ argue that the water consumption should be assessed independent of the state of the local or regional water resources. This means that a certain (volumetric) water footprint should not be handled differently in water scarce regions compared to areas of abundant water resources. Scientists from the LCA‐based tradition propose using characterization factors given by the present state of the water resources, which make water consumption in water‐scarce areas more problematic.

As a support to the use of characterization factors (“impact factors”), we would like to reflect over the argument that “water is a global resource.”^44^ Globally there are vast volumes of freshwater available. Based on the numbers published by Shiklomanov,^46^ the long‐term average volume of renewable freshwater resources is 42 700 km^3^ per year. This volume provides ≈5800 m^3^ of water per capita per year. This is much higher than the threshold values for water stress, starting when the volumes are lower than 1700 m^3^ per capita per year, or water scarcity when the volumes are lower than 1000 m^3^ per capita per year.^47^ The problem is the uneven distribution of water in space and time. As such, we support the view that the severity of the impacts of elevated water losses must be assessed in the light of the state of the local or regional water resources.

Different sets of characterization factors are available,^43^ and the methodology for characterization is under development.^24^ demonstrated the use of characterization factors on two Norwegian hydropower plants with use of the LCA‐based tool SimaPro ver. 8.0.4^48^ linked with the EcoInvent 3.1 database. This study used characterization factors developed for countries, or coarser spatial resolution, while ref. 26 related the water footprint to water scarcity at river basin level.^16^ made their assessment of impacts based in a description of local/regional aridity.

The latest version of the ISO Water Footprint standard^49^ describes an approach that takes into account the positive effect of increased availability of water due to regulation, based on methodology outlined by Pfister and Baumann.^50^ Details on the calculation are provided in the illustrative examples supporting ISO 14044.^49^ Scherer and Pfister^18^ applied a variant of the methodology by Pfister and Baumann.^50^ Based on their dataset of close to 1500 hydropower plants, close to half of these projects obtain negative water scarcity footprints, which means that they alleviate rather than worsen water scarcity. Despite the improvements in methodology to capture the impacts of the water consumption, as well as the positive effect of the improved availability, the present methodologies need further refinements. Similar to the impact, the societal value of the increased availability will depend on the local or regional context.

The purpose of reservoirs is to overcome the natural hydrological variability in order to secure access to water in dry periods. The impacts of the elevated water consumption must be compared with the positive effect of the regulation, which is increased availability of water in periods the natural runoff is lower than the demand. The redistribution of water in time, sometimes also in space as the regulation might enable transfer of water, will create new winners and losers of water in areas of limited resources. The geographical reallocation of the impacts and benefits caused by the reservoir should more precisely be described. The question to address is the trade‐off between annually reduced runoff and increased availability in periods with little water, i.e., when the water has a high value to the society, or to a set of water users. As one example,^33^ reported very large social and economic benefits of the improved availability of water due to the regulation of Lake Nasser, higher than the costs of the extremely high evaporative losses from regulated lake.

A starting point for an improved description could be to redesign some of the large number of indices and indicators available for the assessment of water scarcity/stress and aridity (e.g., refs. 51, 52, such as the well‐established Falkenmark indicator,^47^ Aridity Index,^53^ Global monthly water scarcity,^54^ and the recently published Water depletion index.^55^ One weakness of these indices is the poor handling in representing infrastructure such as reservoirs on the proper spatial and temporal resolution, and the variable water needs, for instance in the agricultural sector. One way forward could also be to adopt elements from the Building Block Methodology,^56^ originally developed for the purpose of setting environmental flows, but in a pilot study modified to assess water needs across sectors.^57^

### 3.5 The Quality of the Energy Provided

All energy systems need ancillary services in order to keep the balance between the energy production and consumption (load) stable and avoid blackouts. In energy systems with a large share of intermittent sources, such as wind and solar power, the need for such balancing services is large. According to Edenhofer et al.,^3^ hydropower is also the only large‐scale provider of energy storage, and is very important in many energy systems.^58^ In contrast to those technologies exclusively producing base‐load power, such as nuclear and coal‐fired power plants, and the intermittent sources, regulated hydropower can provide a broader spectrum of energy services. The role of hydropower in an energy system will depend on the demand pattern, and the composition of energy sources.

Pumped‐storage hydropower plants (PSPs) are built for the specific purpose of meeting peak power demands. In this context, it is relevant to distinguish between those PSPs that are closed‐loop systems, i.e., the same volume of water is circulated in a closed system and those PSPs with some inflow. A closed‐loop PSP will be a net consumer of electricity, because of energy losses in the waterways, turbines, and generators, and the present methodology for calculation of water consumption is not feasible for these types of plants. Those PSPs that receive some natural inflow might produce some net power, if the power produced from the inflow exceeds the power consumed due to pumping. Due to the definition of the water consumption equation, the water consumption will be infinite as the net power approach zero, which appears meaningless.

The published studies on water footprint from hydropower do not discuss the differences in energy quality. The value of peak power can be much higher than base‐load production, seen from a system integrator's point of view. As such, plants designed for peak power production with lower annual production than base load producers should be granted lower water consumption values due to the higher quality of energy they provide.

## 4 Conclusion

This paper reviews published estimates of water consumption from hydropower production and the methodologies applied. Published values range from negative to more than 115 000 m^3^ MWh^−1^, where the high numbers are extremely high when compared to other technologies. Most gross water consumption rates are in the range 5.4–234 m^3^ MWh^−1^, while most net values are in the range 0.2–140 m^3^ MWh^−1^. The lowest values are from cold‐climate regions, such as Norway, Canada, and parts of USA and Russia, or are calculated from run‐of‐the‐river plants with no reservoirs. The highest published numbers are from China, West‐Africa, USA, and Canada, calculated from plants with large reservoir surfaces and high evaporation rates. Net values are often less than 40% of the gross values, sometimes only 1% of the gross water consumption estimates. The extremely wide range in estimates is explained by an inconsistent methodology and the very site‐specific nature of hydropower projects. As such, it is difficult to propose “typical values” for water consumption from hydropower production.

The scientific challenges of allocation from multipurpose reservoirs and the spatial assignments in river basins with several hydropower plants still remain unresolved, which affect the results dramatically. Those studies allocating the water losses between multiple purposes conclude that the allocation significantly reduces the water consumption assigned to the power production. There is limited awareness and no clear procedures on the spatial influence of river regulations, as the water losses from the reservoir are in most studies directly assigned to the closest power plant. This implies that very high numbers are assigned to reservoir‐based plants and very low values to run‐of‐the‐river plants, even though the run‐of‐the‐river plants also benefit from the regulation.

A number of critical shortcomings have been identified in the methodology and this paper points out directions of research in order to develop a consistent, extended, and fair methodology for the calculation of water consumption from hydropower projects. We propose the following steps to be taken in order to establish such a methodology;

- Scientific agreement on the use of net or gross evaporation rates as input to water consumption calculation should be reached.
- In order to assess the real net effect, it should be considered to take into account also other parts of the regulated system affected by the changes in water flow, as well as the operation of the reservoirs.
- Methodology for allocation from multipurpose reservoirs should be further developed and tested.
- Methodology to handle the spatial complexity of hydropower regulations should be developed. Research is also needed to define the proper spatial boundaries, i.e., the influence area, in order to capture the full effect (positive and negative) of the regulation.
- All impacts of the regulation should be taken into account, i.e., both the possible negative effect of the reduced downstream flow, as well as the positive effect of increased availability.
- A metrics for describing the quality of energy service provided should be considered included in the calculations.

## Conflict of Interest

The authors declare no conflict of interest.
